# Triglyceride glucose index as a predictor of mortality in middle-aged and elderly patients with type 2 diabetes in the US

**DOI:** 10.1038/s41598-023-43512-0

**Published:** 2023-09-30

**Authors:** Mengjie Zhao, Mengli Xiao, Qin Tan, Fang Lu

**Affiliations:** 1 NMPA Key Laboratory for Clinical Research and Evaluation of Traditional Chinese Medicine, Xiyuan Hospital, China Academy of Chinese Medicine Sciences, 1 Xiyuan Caochang, Haidian District, Beijing, 100091 China; 2 National Clinical Research Center for Chinese Medicine Cardiology, Xiyuan Hospital, China Academy of Chinese Medicine Sciences, 1 Xiyuan Caochang, Haidian District, Beijing, 100091 China; 3 Graduate School of Beijing University of Chinese Medicine, 11 North 3rd Ring East Road, Chaoyang District, Beijing, 100029 China

**Keywords:** Endocrinology, Medical research, Risk factors

## Abstract

Despite a wealth of research linking the triglyceride glucose index (TyG index) to metabolic diseases. However, little evidence links the TyG index to all-cause or CVD mortality in middle-aged and elderly individuals with type 2 diabetes (T2D). This study analyzed data from 2998 patients with T2D who participated in the National Health and Nutrition Examination Survey (NHANES) between 1999 and 2018. The TyG index and mortality in middle-aged and elderly T2D patients were investigated using Cox regression models. The nonlinear association between the TyG index and mortality can be understood with the help of a restricted cubic spline (RCS). During a median follow-up period of 82 months, 883 fatalities were observed from all causes and 265 from CVD. The TyG index was found to have a U-shaped relationship with all-cause and CVD mortality in T2D, with cutoffs of 8.95 and 9, respectively, according to the RCS. After controlling for other factors, an increase of 1 unit in the TyG index was related to an increase of 33% in all-cause mortality and 50% in CVD mortality when TyG was ≥ 8.95 and 9. When TyG < 8.95 and 9, with the change in the TyG index, the change in all-cause and CVD death was insignificant. Patients with T2D who are middle-aged or older, especially elderly patients, have higher TyG levels associated with increased mortality. In middle-aged and elderly patients with T2D, the TyG index may predict the probability of death from any cause and death from CVD.

## Introduction

Type 2 diabetes (T2D) is a metabolic disease with a relative or absolute insulin deficiency as the pathophysiology and is influenced by several factors^1^. T2D has steadily increased during the past few years^2^. There are already 537 million people with diabetes, which is anticipated to rise to 783 million by 2045, according to epidemiological data^3^. Diabetes has grown to be a significant financial burden for people, families, and nations as one of the top causes of death in the middle-aged and older population^4^.

Insulin resistance (IR) is essential in developing T2D, especially in skeletal muscle and the liver, as an important mechanism leading to T2D^5^. Although the hyper insulin-normoglycemic clamp assay is widely regarded as the gold standard for evaluating IR^6^, its complexity and high cost make it unsuitable for large-scale clinical investigations. After that, the homeostatic model assessment of insulin resistance (HOMA-IR) was proposed to quantify IR using fasting glucose and insulin levels indirectly^7^. However, its clinical application is limited by insulin measurement requirements, high test costs, and poor reproducibility. According to research, the serum triglyceride-glucose product index (TyG index) has shown promise as a potential IR alternative^8^. The TyG index has been demonstrated to have a strong relationship with IR in studies using the hyper insulin-normal glucose clamp method^9^. Because of its ease of use, low cost, and high reproducibility, the TyG index has gained popularity in investigations of T2D and cardiovascular disease (CVD).

TyG has been shown in various studies in the past few years to be a useful tool in the risk assessment and diagnosis of diabetes mellitus^10–14^. The TyG index has proven useful in determining the prognosis of patients with CVD and T2D in addition to its superior IR detection ability^15–19^. However, the TyG index's correlation with T2D patients' mortality risk has not been investigated. This study aims to provide population epidemiological evidence that the TyG index is associated with an increased risk of death from all causes and CVD in middle-aged and elderly T2D patients.

## Methods

### Study population

**Figure 1:**
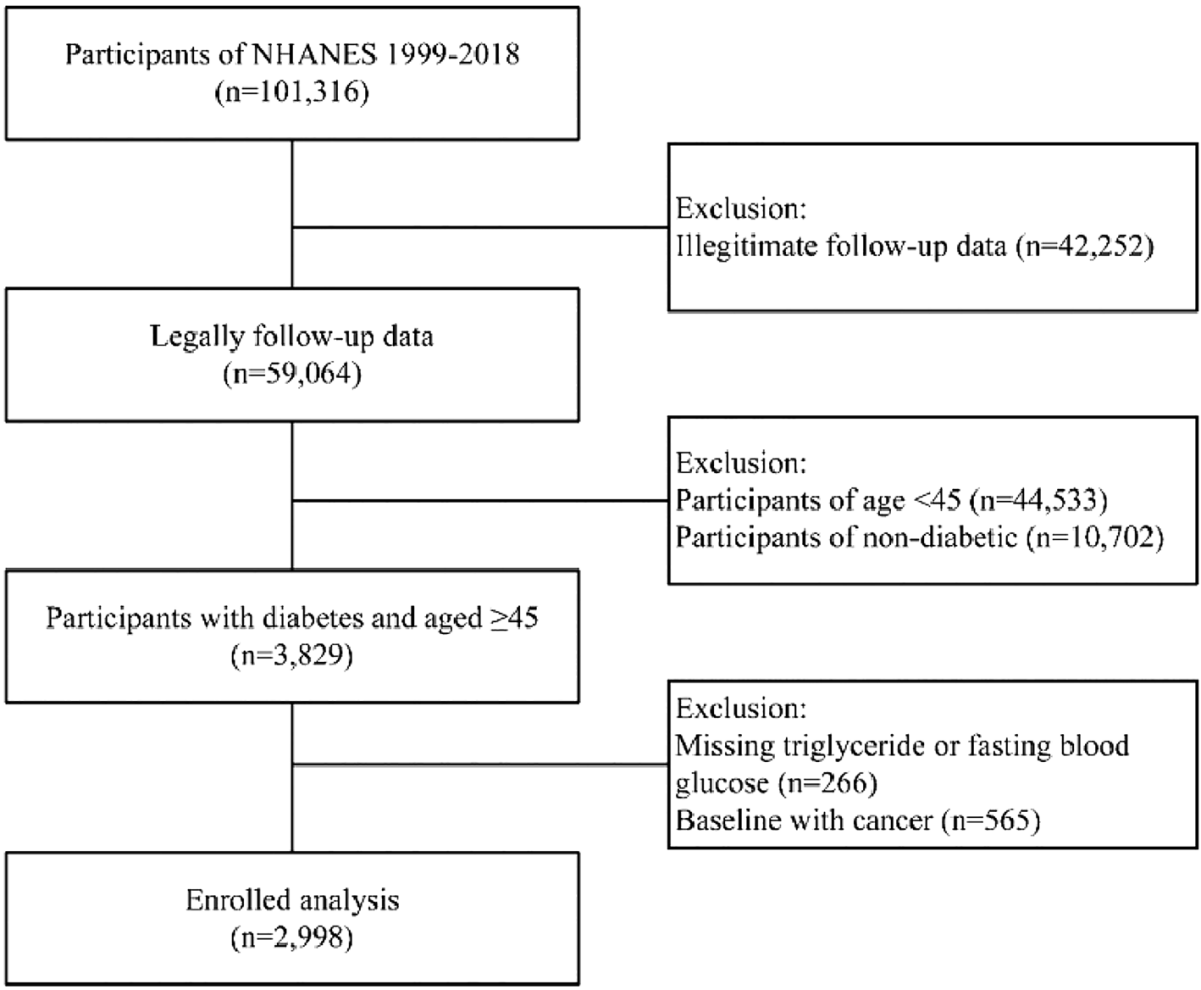
Flowchart of research subject screening.

The participants were culled from the National Health and Nutrition Examination Survey (NHANES). A nationally representative US population survey is performed every two years. It uses a sophisticated multistage probability sampling design to capture the nation's health and nutrition data^20^. The National Death Index (NDI) may be accessed through the database, enabling death records to be tracked prospectively. The NHANES survey was given the go-light by the ethics review committee. A written informed consent form was signed by all of the participants. This study used legitimate follow-up data from 10 survey cycles pooled by NHANES from 1999 to 2018. All participants aged < 45, nondiabetic, and not receiving fasting glucose measurement and triglyceride testing were excluded. In addition, participants with comorbid cancer at baseline were also excluded. Figure 1 depicts the flowchart for selecting participants for the research population. A final total of 2998 participants were included.

### Definition of diabetes mellitus and TyG

Diabetes can be diagnosed if one of the following conditions is met: self-reported diagnosis of diabetes, use of insulin or oral hypoglycemic drugs, fasting blood glucose levels ≥ 7.0 mmol/L, random blood glucose or two-hour oral glucose tolerance test (OGTT) blood glucose levels ≥ 11.1 mmol/L, or glycated hemoglobin A1c (HbA1c) levels ≥ 6.5%^21^. The TyG index is calculated as TyG = Ln [fasting triglycerides (mg/dL) × fasting glucose (mg/dL)/2]^9,22^.

### Definition of all-cause and cardiovascular death

To determine participant survival status, this study used the NHANES Associated Mortality File for 1999–2018. Specific causes of death in the death files were based on the International Classification of Diseases (10th revision, ICD-10). Cardiovascular deaths (including rheumatic heart disease, hypertensive heart disease, ischemic heart disease, acute myocardial infarction, pericardial disease, and acute myocarditis and heart failure) correspond to disease codes I00–I09, I11, I13, and I20–I51. Detailed information can be found in the deaths available in the Underlying and Multiple Cause of Death Codes (CDC).

### Covariates

The following factors were chosen for examination based on reviews of relevant literature and observations made in clinical settings^23–25^. The following demographic information was collected: age, gender, education level, poverty impact ratio, and race/ethnicity. Answers to questions about smoking, drinking, glucose-lowering medicine use, omega-3 supplements, glucocorticoid drugs, hypertension, hyperlipidemia, coronary heart disease (CHD), heart failure (HF), heart attack, stroke, and angina were gleaned from “yes” or “no” choices on the questionnaire. BMI was determined using the following formula: BMI = weight (kg)/height^2^ (m^2^)^26^. BMI was divided into four groups: obesity (≥ 30), overweight (25 ≤ BMI < 30), normal weight (18.5 ≤ BMI < 25), and low weight (< 18.5)^27^. The Geriatric Nutritional Index (GNRI) was determined using the following formula: GNRI = (14.89 × albumin (g/dL)) + (41.7 × (body weight/ideal body weight))^28^. Biochemical data were obtained from laboratory test data, including HbA1c, low-density lipoprotein cholesterol (LDL-C), total cholesterol (TC), and albumin.

### Statistical analysis

Categorical data are reported as weighted percentages, whereas nonnormal continuous variables are expressed as weighted medians (interquartile spacing, IQR). To characterize the connection between the TyG index and both all-cause and CVD mortality, this study employed a generalized summation model-based smoothed curve fitting approach. Segmented linear regression was used for nonlinear relationships to calculate effect values for each interval separately. The log-likelihood ratio test was used to compare the regular linear regression model to the segmented linear regression model and to determine whether a turning point existed (a turning point was considered to exist at *P* < 0.05). The turning point value was determined using the maximum likelihood method. Cox proportional risk models were used to analyze the association between the TyG index and mortality. Subgroups stratified by age, sex, HbA1c, BMI, and smoking status were examined for differences in the connection between the TyG index and the risk of death in individuals with T2D. The discriminatory power of the TyG index for all-cause and cardiovascular mortality was analyzed using time-dependent receiver operating characteristic curves (ROCs). To increase statistical power and reduce the possibility of bias due to missing data, we employed several interpolations to fill in missing covariate values^29^. A sensitivity analysis is conducted to ensure the test results' validity. The statistical analysis procedure considered sample weights according to NHANES data analysis guidelines. Two-tailed *P* values < 0.05 were considered significant. All statistical tests were performed in R 4.1.2.

### Ethics approval and consent to participate

The study received approval from the National Centre for Health Statistics Research Ethics Review Board.

## Results

A total of 2998 study subjects aged 45 years and older were included, of which 1594 (53.17%) were men. Participants' median age was 64 (IQR: 45–85). Table 1 details the baseline parameters, stratified by TyG index. Of these, 1190 (39.69%) participants had no high school or higher education, 1702 (56.77%) used glucose-lowering medications, 1442 (48.10%) were obese, 1549 (51.67%) smoked, and 1967 (65.61%) consumed alcohol. Patients with T2D Patients with T2D in the TyG > 9.65 group were younger and more likely to be obese and smokers (*p* < 0.05). Increases in the TyG index were associated with an elevated risk of hyperlipidemia (*p* < 0.001) but not CHD or stroke (*p* > 0.05). Increases in the TyG index were associated with increases in TC, LDL-C, and HbA1c (*p* < 0.001). We recorded 883 fatalities during the course of a median of 82 months of observation, 265 of which were attributable to CVD. Additionally, we compared the baseline characteristics of deceased and surviving patients in the inclusion group. Participants who were still alive were younger, more commonly male, more educated, and more likely to be obese, drinkers, and nonsmokers than those who had died. Living participants also had a lower prevalence of HF, CHD, angina, heart attack, stroke, and hypertension. Deceased patients had higher TyG indices (*p* < 0.001) compared with living T2D patients (Supplementary Table 1).

**Table 1:** Baseline features of middle-aged and older people with T2D in the NHANES.

	Total	Triglyceride-glucose index				*P*-value
		TyG ≤ 8.72	8.72 < TyG ≤ 9.15	9.15 < TyG ≤ 9.65	TyG > 9.65	
N	2998	754	740	757	747	
Age, years, median (IQR)	64.00 (45.00, 85.00)	65.50 (58.20, 74.00)	65.00 (58.00, 72.00)	64.00 (56.00, 72.00)	61.00 (54.00, 69.00)	< 0.001
Gender, n (%)						0.105
Male	1594 (53.17%)	400 (53.05%)	401 (54.19%)	376 (49.67%)	417 (55.82%)	
Education status, n (%)						0.003
Less than high school	1190 (39.69%)	268 (35.54%)	292 (39.46%)	303 (40.03%)	327 (43.78%)	
High school or equivalent	680 (22.68%)	191 (25.33%)	144 (19.46%)	188 (24.83%)	157 (21.02%)	
College or above	1128 (37.63%)	295 (39.12%)	304 (41.08%)	266 (35.14%)	263 (35.21%)	
Race, n (%)						< 0.001
Non-Hispanic white	635 (21.18%)	95 (12.60%)	143 (19.32%)	172 (22.72%)	225 (30.12%)	
Non-Hispanic black	311 (10.37%)	65 (8.62%)	84 (11.35%)	82 (10.83%)	80 (10.71%)	
Mexican American	1005 (33.52%)	210 (27.85%)	258 (34.86%)	266 (35.14%)	271 (36.28%)	
Other	1047 (34.92%)	384 (50.93%)	255 (34.46%)	237 (31.31%)	171 (22.89%)	
PIR level, n (%)						0.105
≤ 1.0	683 (22.78%)	155 (20.56%)	155 (20.95%)	177 (23.38%)	196 (26.24%)	
1.1–3.0	1412 (47.10%)	360 (47.75%)	348 (47.03%)	365 (48.22%)	339 (45.38%)	
> 3.0	903 (30.12%)	239 (31.70%)	237 (32.03%)	215 (28.40%)	212 (28.38%)	
BMI, kg/m^2^, n (%)						< 0.001
Normal weight	514 (17.14%)	187 (24.80%)	128 (17.30%)	99 (13.08%)	100 (13.39%)	
Low weight	16 (0.53%)	5 (0.66%)	4 (0.54%)	4 (0.53%)	3 (0.40%)	
Overweight	1026 (34.22%)	254 (33.69%)	268 (36.22%)	251 (33.16%)	253 (33.87%)	
Obesity	1442 (48.10%)	308 (40.85%)	340 (45.95%)	403 (53.24%)	391 (52.34%)	
Smoking, n (%)						0.026
Yes	1549 (51.67%)	358 (47.48%)	377 (50.95%)	406 (53.63%)	408 (54.62%)	
Alcohol intake, n (%)						0.657
Yes	1967 (65.61%)	494 (65.52%)	498 (67.30%)	486 (64.20%)	489 (65.46%)	
HF, n (%)						0.071
Yes	302 (10.07%)	93 (12.33%)	62 (8.38%)	71 (9.38%)	76 (10.17%)	
CHD, n (%)						0.853
Yes	319 (10.64%)	80 (10.61%)	73 (9.86%)	82 (10.83%)	84 (11.24%)	
Angina, n (%)						0.934
Yes	220 (7.34%)	54 (7.16%)	55 (7.43%)	59 (7.79%)	52 (6.96%)	
Heart attack, n (%)						0.527
Yes	349 (11.64%)	98 (13.00%)	85 (11.49%)	80 (10.57%)	86 (11.51%)	
Stroke, n (%)						0.283
Yes	257 (8.57%)	76 (10.08%)	60 (8.11%)	66 (8.72%)	55 (7.36%)	
Hypertension, n (%)						0.037
Yes	1997 (66.61%)	517 (68.57%)	486 (65.68%)	524 (69.22%)	470 (62.92%)	
Hyperlipidemia, n (%)						< 0.001
Yes	1789 (59.67%)	395 (52.39%)	433 (58.51%)	479 (63.28%)	482 (64.52%)	
Hypoglycemic drugs use, n (%)						0.116
Yes	1702 (56.77%)	429 (56.90%)	420 (56.76%)	406 (53.63%)	447 (59.84%)	
Total cholesterol, mg/dl, median (IQR)	183.00 (75.00, 460.00)	166.00 (142.00, 195.00)	175.00 (152.00, 206.00)	186.00 (161.00, 216.00)	204.00 (176.00, 242.00)	< 0.001
LDL cholesterol, mg/dl, median (IQR)	104.00 (15.00, 370.00)	94.00 (72.00, 117.00)	101.00 (79.00, 127.00)	108.00 (83.00, 134.00)	118.00 (89.00, 149.50)	< 0.001
HbA1c, %, median (IQR)	6.80 (3.50, 18.00)	6.30 (5.80, 6.90)	6.60 (6.10, 7.40)	6.80 (6.20, 7.80)	7.90 (6.90, 9.70)	< 0.001
Fasting blood glucose, median (IQR)	7.66 (6.66, 9.77)	6.50 (5.65, 7.33)	7.33 (6.61, 8.49)	7.99 (7.11, 9.66)	11.12 (8.62, 14.69)	< 0.001
Random blood glucose, median (IQR)	7.33 (6.37, 9.44)	6.27 (5.33, 6.99)	7.05 (6.27, 8.16)	7.66 (6.73, 9.33)	10.66 (8.22, 14.04)	< 0.001

T2D, Type 2 diabetes; TyG, triglyceride-glucose; IQR, interquartile range; PIR, poverty income ratio; BMI, body mass index; HF, heart failure; CHD, coronary heart disease; LDL, low density lipoprotein; HbA1c, glycated hemoglobin A1c.

**Figure 2:**
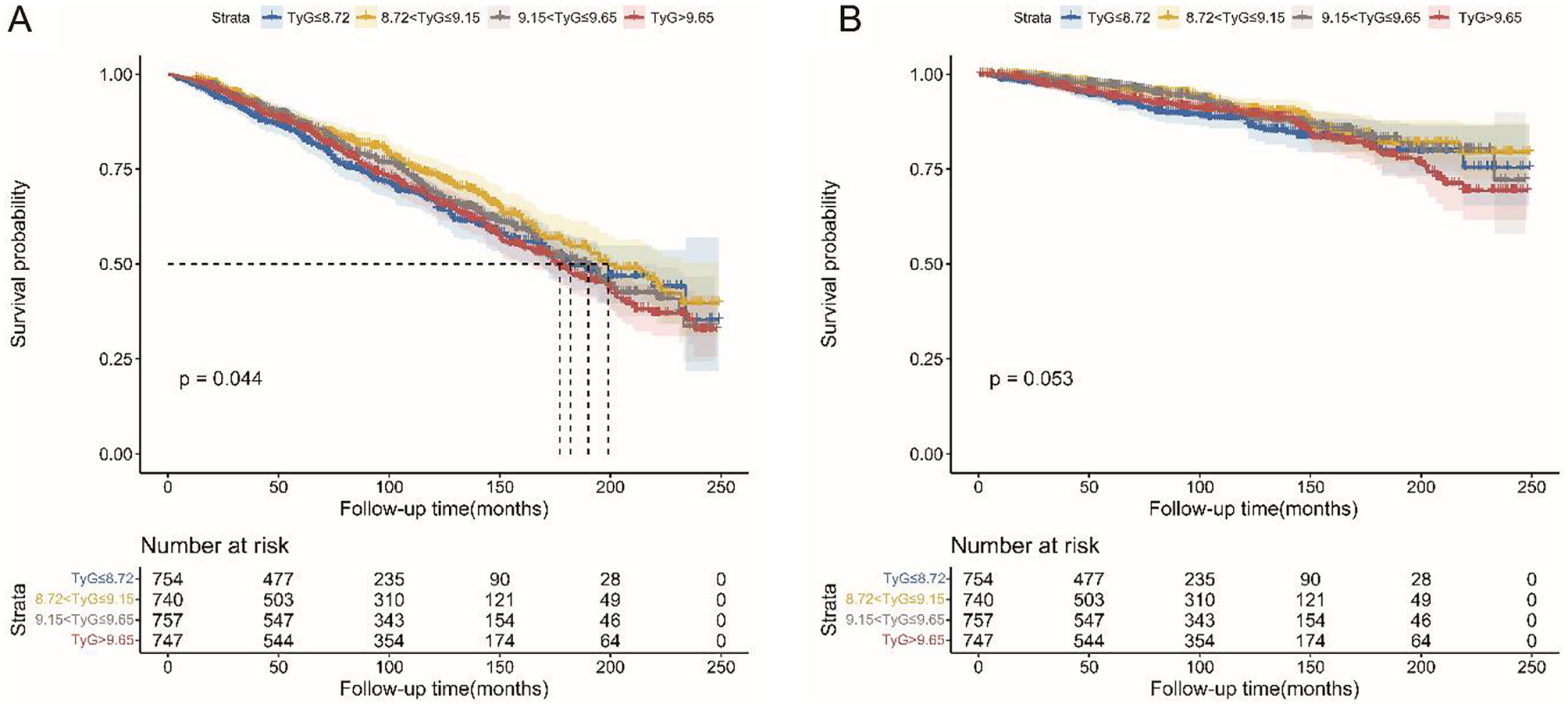
Kaplan‒Meier survival rates for middle-aged and elderly T2D patients in different TyG index groups. (**A**) All-cause mortality; (**B**) CVD mortality.

The relative risk of all-cause and CVD mortality in relation to the TyG index is shown in Fig. 2. Patients with T2D who reported a TyG > 9.65 had a substantially greater risk of all-cause death than those who reported a TyG ≤ 8.72 (log-rank *p* = 0.044). In contrast, no significant change in CVD mortality was observed (log-rank *p* = 0.053).

**Figure 3:**
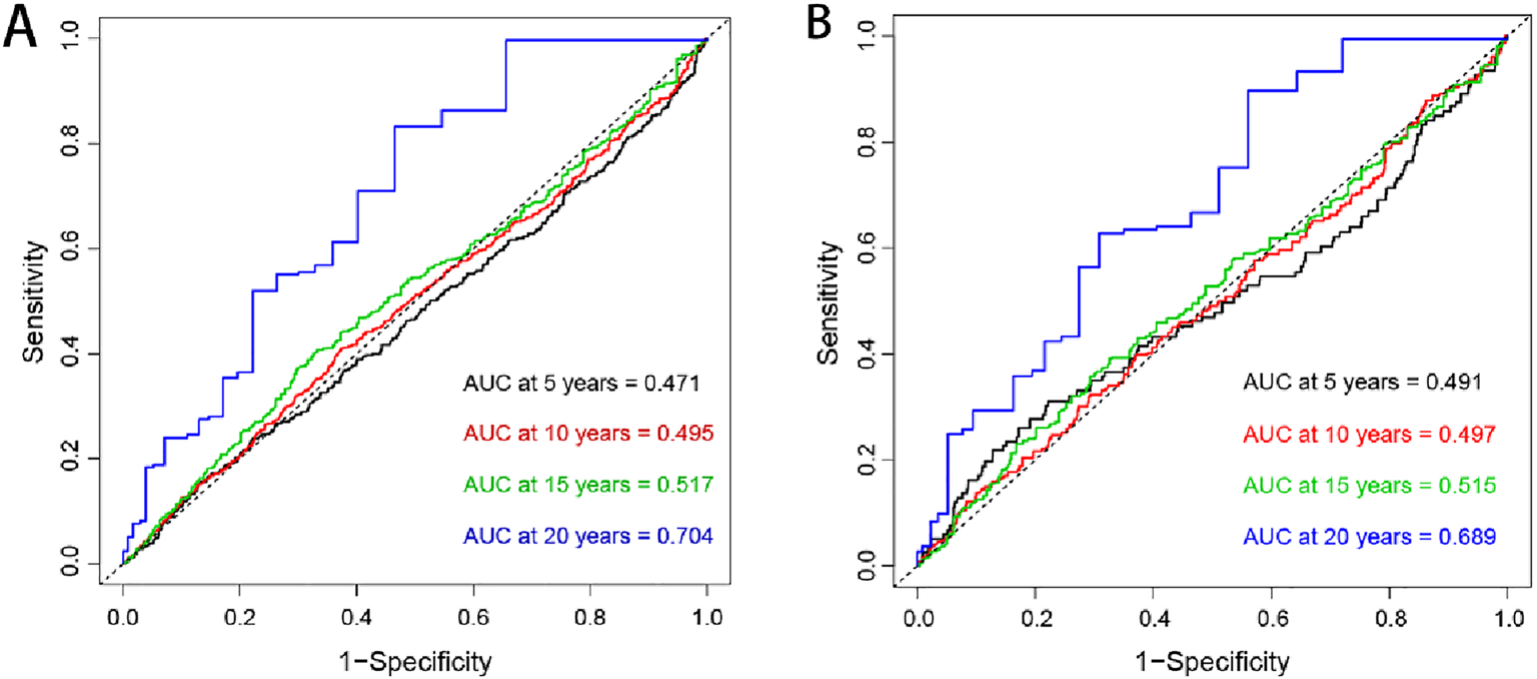
Time-dependent receiver operating characteristic curve analysis of TyG index distinguishing between all-cause (**A**) and cardiovascular mortality risk (**B**) at different time points.

**Figure 4:**
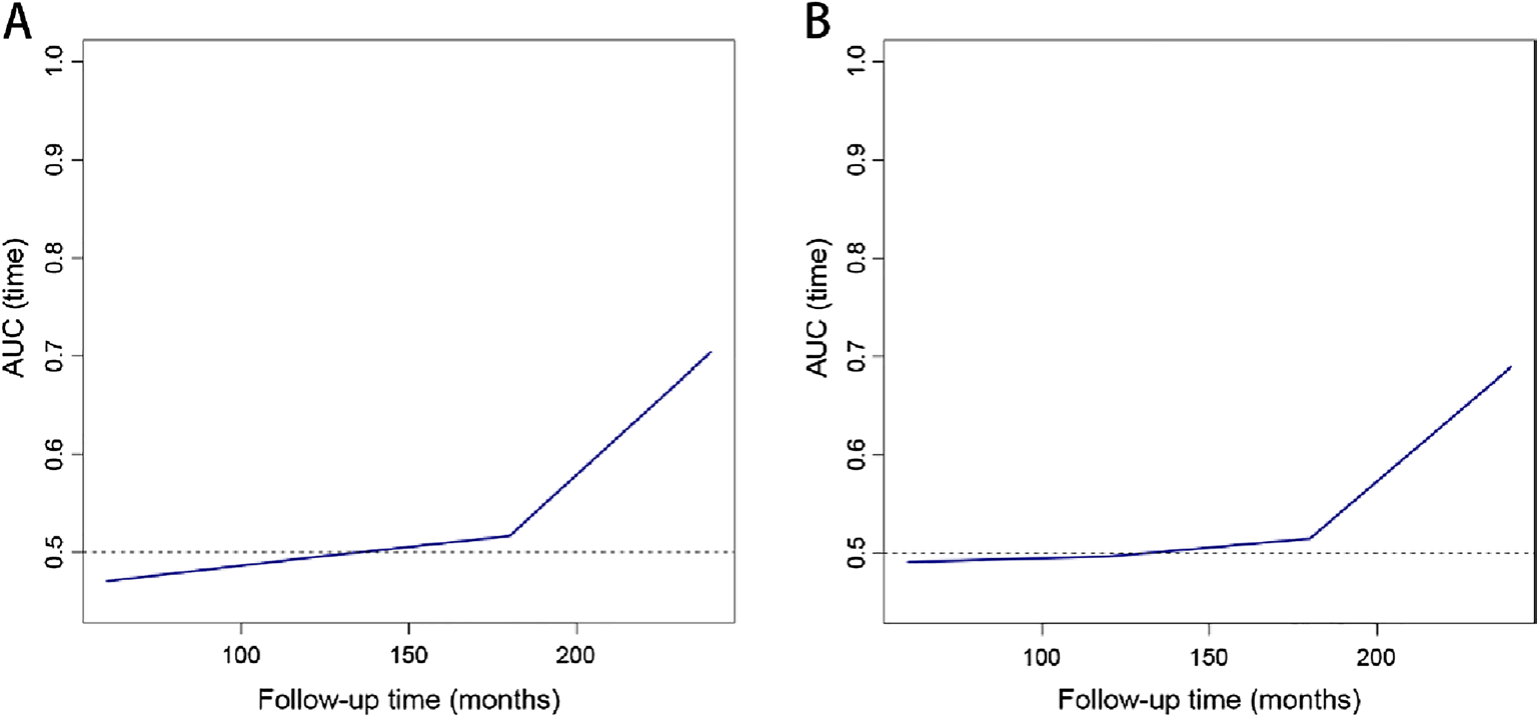
Comparison of AUC for TyG index to differentiate between all-cause (**A**) and cardiovascular mortality (**B**) at different periods.

Time-dependent ROC curve analyses were performed to analyze the risk of all-cause and cardiovascular mortality identified by the TyG index at different time points. The AUC of the TyG index for identifying the risk of all-cause mortality was 0.471 at 5 years, 0.495 at 10 years, 0.517 at 15 years, and 0.704 at 20 years. In addition, the AUC of the TyG index identifying the risk of cardiovascular death was 0.491 at 5 years, AUC was 0.497 at 10 years, AUC was 0.515 at 15 years, and AUC was 0.689 at 20 years. The above results are shown in Fig. 3. In this study, we found that the AUC of the TyG index identifying both all-cause and cardiovascular mortality risk increased over time (Fig. 4).

The correlation between the TyG index and mortality was further investigated using Cox analysis (Table 2). Using the TyG ≤ 8.72 group as a reference, the remaining three groups (8.72 < TyG ≤ 9.15, 9.15 < TyG ≤ 9.65, and TyG > 9.65) had hazard ratios [HRs (95% CI)] for all-cause mortality of 0.80 (0.65, 0.98), 0.88 (0.72, 1.08), and 1.14 (0.91, 1.41) (*P* for trend = 0.174) and HRs (95% CI) for CVD mortality of 0.69 (0.48, 1.00), 0.72 (0.50, 1.04), and 1.04 (0.70, 1.53) (*P* for trend = 0.833), respectively, after adjusting for age, sex, race/ethnicity, education level, PIR, BMI, smoking status, alcohol consumption status, CHD, HF, heart attack, stroke, angina, hypertension, hyperlipidemia, glucose-lowering medication use, HbA1c, LDL-C, and TC (model 3). Mortality from all causes and CVD were both lower in the 8.72 < TyG ≤ 9.15 group than in the TyG ≤ 8.72 group (*p* < 0.05) (Table 2).

**Table 2:** Multivariate Cox regression analysis of the TyG index and all-cause and CVD mortality.

	No. deaths (%)	Crude	Model 1	Model 2	Model 3
		HR (95% CI), *p* value	HR (95% CI), *p* value	HR (95% CI), *p* value	HR (95% CI), *p* value
All-cause mortality					
TyG index					
≤ 8.72	203 (26.92)	1 (Ref)	1 (Ref)	1 (Ref)	1 (Ref)
8.72–9.15	186 (25.14)	0.77 (0.63, 0.94), 0.011	0.76 (0.62, 0.93), 0.007	0.79 (0.65, 0.97), 0.022	0.80 (0.65, 0.98), 0.030
9.15–9.65	229 (30.25)	0.88 (0.73, 1.06), 0.175	0.88 (0.73, 1.07), 0.200	0.87 (0.72, 1.06), 0.161	0.88 (0.72, 1.08), 0.213
> 9.65	265 (35.48)	0.97 (0.80, 1.16), 0.718	1.09 (0.91, 1.32), 0.349	1.14 (0.94, 1.38), 0.172	1.14 (0.91, 1.41), 0.250
*P* for trend		0.776	0.108	0.07	0.174
Cardiovascular mortality					
TyG index					
≤ 8.72	67 (8.89)	1 (Ref)	1 (Ref)	1 (Ref)	1 (Ref)
8.72–9.15	52 (7.03)	0.65 (0.46, 0.94), 0.022	0.64 (0.44, 0.92), 0.015	0.70 (0.48, 1.01), 0.055	0.69 (0.48, 1.00), 0.052
9.15–9.65	62 (8.19)	0.72 (0.51, 1.01), 0.060	0.71 (0.50, 1.00), 0.051	0.74 (0.52, 1.05), 0.091	0.72 (0.50, 1.04), 0.081
> 9.65	84 (11.24)	0.93 (0.67, 1.28), 0.644	1.03 (0.74, 1.43), 0.851	1.12 (0.80, 1.57), 0.506	1.04 (0.70, 1.53), 0.857
*P* for trend		0.943	0.575	0.38	0.833

CVD, cardiovascular disease; HR, hazard ratio; CI, confidence interval; Ref, reference.

Crude: unadjusted;

Model 1: corrected for age, sex, education level, PIR and BMI;

Model 2: Model 1 + race/ethnicity, smoking status, alcohol consumption status, CHD, HF, heart attack, stroke and angina;

Model 3: Model 2 + hypertension, hyperlipidemia, glucose-lowering medication use, HbA1c, LDL-C and TC.

We performed sensitivity analyses by eliminating missing values at baseline, excluding people taking omega-3 supplements and glucocorticoid medications, and adjusting for the Geriatric Nutritional Index (GNRI), to ensure the reliability of the results (Supplementary Tables 2–4). The results showed that the risk of all-cause mortality [HR (95% CI), 0.70 (0.53, 0.93), 0.80 (0.65, 0.98) and 0.81 (0.66, 0.99)] was significantly lower in all three sensitivity-analyzed groups of 8.72 < TyG ≤ 9.15 compared to the group with the lowest TyG index (TyG ≤ 8.72). However, we found no statistically significant correlation between a high TyG index and mortality from cardiovascular causes.

**Figure 5:**
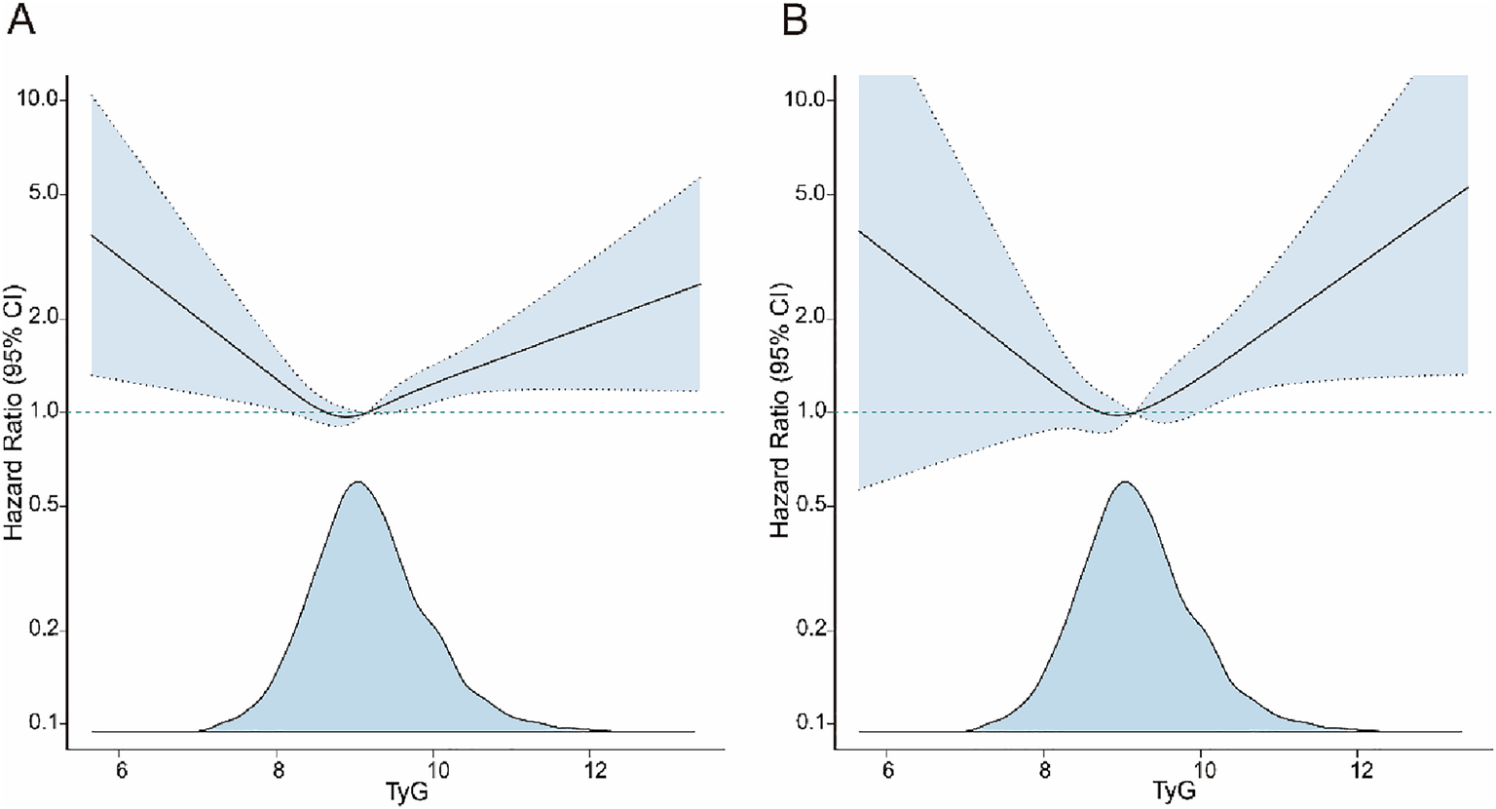
Nonlinear analysis of T2D mortality and TyG index in middle-aged and older adults.

Additionally, we used multivariate adjusted regression to check for a connection between the TyG index and mortality (Fig. 5). The TyG index was found to have a U-shaped relationship with both all-cause and CVD mortality (Fig. 5A and Fig. 5B, respectively) in middle-aged and elderly T2D patients, as determined by an RCS analysis. A likelihood ratio test confirmed the nonlinear link between the TyG index and all-cause and CVD mortality (*p* = 0.001 and 0.015) (Table 3). Analysis of threshold effects showed that the critical point for all-cause mortality was 8.95, and for CVD mortality, it was 9. After controlling for other factors, an increase of 1 unit in the TyG index was linked with a 33% and 50% increase in all-cause and CVD mortality [HR (95% CI), 1.33 (1.13, 1.56) and 1.50 (1.12, 2.00)], respectively, at TyG ≥ 8.95 and 9. When TyG < 8.95 and 9, the differences in all-cause and CVD mortality were not significant with changes in the TyG index [HR (95% CI), 0.76 (0.55, 1.06) and 0.76 (0.43, 1.33)] (Table 3).

**Table 3:** Threshold effect analysis of the TyG index with all-cause and CVD mortality.

	All-cause mortality		CVD mortality	
	HR (95% CI)	*P* value	HR (95% CI)	*P* value
Cutoff value	8.95		9.00	
< Cutoff value	0.76 (0.55, 1.06)	0.1057	0.76 (0.43, 1.33)	0.3354
≥ Cutoff value	1.33 (1.13, 1.56)	< 0.001	1.50 (1.12, 2.00)	0.0062
Likelihood ratio test	0.001		0.015	

Both linear regressions were adjusted for age, sex, education level, race/ethnicity, PIR, smoking status, drinking status, BMI, CHD, HF, heart attack, stroke, angina, hypertension, hyperlipidemia, glucose-lowering medication use, HbA1c, LDL-C, and TC.

To further examine the difference between the high TyG index group (≥ 8.95 or 9) and the low TyG index group (< 8.95 or 9), we conducted a subgroup study of middle-aged and elderly individuals with T2D. The analysis results, which were stratified by age, sex, HbA1c, BMI, and smoking status, are presented in Table 4. At TyG above the threshold of 8.95, age ≥ 65 years (HR 1.28; 95% CI 1.03, 1.60), female sex (HR 1.34; 95% CI 1.05, 1.71), HbA1c ≥ 6.5% (HR 1.28; 95% CI 1.08, 1.52), BMI ≥ 25 kg/m^2^ (HR 1.32; 95% CI 1.11, 1.57), and smoking (HR 1.29; 95% CI 1.06, 1.57) had a significant positive correlation with all-cause mortality. At TyG above threshold 9, the association between the TyG index and CVD mortality was influential in males (HR 1.64; 95% CI 1.14, 2.35), HbA1c ≥ 6.5% (HR 1.44; 95% CI 1.06, 1.97), BMI ≥ 25 kg/m^2^ (HR 1.50; 95% CI 1.09, 2.06), and smoking (HR 1.77; 95% CI 1.23, 2.54). Notably, when TyG > 9, in the subgroup aged ≥ 65, the increase in CVD mortality with increasing TyG index was insignificant (HR 1.49; 95% CI 1.00, 2.22), although the 95% CI did not cross 1.

**Table 4:** Subgroup analysis of TyG index and mortality in patients with T2D.

Cutoff value	N	All-cause mortality		*P* for log likelihood ratio test	CVD mortality		*P* for log likelihood ratio test
		HR (95% CI), *p* value			HR (95% CI), *p* value		
		< 8.95	≥ 8.95		< 9.00	≥ 9.00	
Age, years							
≥ 65	1444	0.75 (0.51, 1.10), 0.135	1.28 (1.03, 1.60), 0.027	0.007	0.85 (0.44, 1.63), 0.621	1.49 (1.00, 2.22), 0.050	0.065
< 65	1554	0.90 (0.46, 1.75), 0.750	1.15 (0.92, 1.44), 0.215	0.369	0.48 (0.14, 1.63), 0.238	1.18 (0.76, 1.81), 0.458	0.144
Gender							
Male	1594	0.87 (0.57, 1.34), 0.535	1.18 (0.96, 1.46), 0.116	0.047	0.78 (0.35, 1.70), 0.526	1.64 (1.14, 2.35), 0.007	0.021
Female	1404	0.59 (0.34, 1.01), 0.056	1.34 (1.05, 1.71), 0.017	0.018	0.69 (0.26, 1.83), 0.457	1.19 (0.72, 1.98), 0.498	0.249
HbA1c, %							
< 6.5	1115	0.92 (0.58, 1.46).0.723	1.18 (0.74, 1.87), 0.495	0.401	1.15 (0.49, 2.70), 0.740	1.21 (0.47, 3.12), 0.698	0.768
≥ 6.5	1883	0.65 (0.40, 1.06), 0.085	1.28 (1.08, 1.52), 0.004	0.009	0.59 (0.26, 1.32), 0.200	1.44 (1.06, 1.97), 0.022	0.026
BMI, kg/m^2^							
< 25	530	0.63 (0.33, 1.20), 0.158	1.40 (0.95, 2.04), 0.086	0.014	0.47 (0.10, 2.22), 0.341	1.74 (0.79, 3.83), 0.170	0.209
≥ 25	2468	0.88 (0.59, 1.32), 0.542	1.32 (1.11, 1.57), 0.002	0.077	0.76 (0.40, 1.43), 0.390	1.50 (1.09, 2.06), 0.013	0.042
Smoking status							
Yes	1549	0.87 (0.56, 1.36), 0.551	1.29 (1.06, 1.57), 0.010	0.021	0.74 (0.35, 1.59), 0.439	1.77 (1.23, 2.54), 0.002	0.012
No	1449	0.57 (0.34, 0.96), 0.033	1.26 (0.97, 1.63), 0.080	0.014	0.67 (0.27, 1.66), 0.388	1.25 (0.78, 2.02), 0.355	0.178

All subgroups were adjusted for age, sex, education level, race/ethnicity, PIR, smoking status, drinking status, BMI, CHD, HF, heart attack, stroke, angina, hypertension, hyperlipidemia, glucose-lowering medication use, HbA1c, LDL-C, and TC. We excluded stratification variables from the model in the analysis of single subgroups.

## Discussion

This study not only confirmed that the TyG index at baseline had the potential to predict both all-cause and CVD mortality in T2D but also brought to light the U-shaped association between TyG levels and mortality. The risk of death was lowest for those with a TyG index in the middle range (8.72–9.15). The high TyG index group (> 9.65) tended to have a worse prognosis, which is easy to understand, as either elevated glucose or elevated lipids can increase the risk of death. Extreme hypoglycemia may explain why those with a low TyG index (≤ 8.72) have a higher risk of dying^30^. After subgroup analysis, it was found that individuals aged ≥ 65, HbA1c ≥ 6.5%, BMI ≥ 25 kg/m^2^, and smoking aged ≥ 65, HbA1c ≥ 6.5%, BMI ≥ 25 kg/m^2^, and smoking had a significant positive correlation between the TyG index and all-cause and CVD mortality when TyG > 8.95 or 9. Interestingly, when TyG > 8.95, women engaged in the study displayed a notable correlation with all-cause death. Furthermore, when TyG > 9, male sex had a notable correlation with CVD-induced mortality.

The TyG index is a straightforward, trustworthy, and valid alternative to HOMA-IR in detecting IR^22^. Long-established major risk factors for diabetes and cardiovascular disorders are high fasting glucose and triglycerides^31–34^. The combined variables of glucose and triglycerides make the TyG index closely associated with related metabolic diseases. Several recent studies have shown that the TyG index improves the prognosis of CVD^35–38^. Similarly, the TyG index's prognostic utility for diabetic patients has been the subject of extensive research. According to studies by Wang et al., patients with diabetes and acute coronary syndrome (ACS) who also had a high TyG index had a noticeably greater risk of cardiac events^39^. In middle-aged and elderly Chinese diabetics, an elevated TyG index increases CVD risk^40^. Tai et al.^41^ observed that the cumulative TyG index independently predicted cardiovascular events in T2D patients.

There is limited research on the association between TyG index and all-cause and cardiovascular disease (CVD) mortality in diabetic patients. Previous studies have observed a U-shaped relationship, with inflection points ranging from 8.5 to 9.52 in the general population^24,42,43^. Our study focused on diabetes patients and found that the TyG index was correlated with mortality thresholds of 8.95 for all-cause mortality and 9 for CVD mortality. Subgroup analysis revealed a strong correlation between the TyG index and all-cause/CVD mortality in individuals over 65. Additionally, there was a positive association between the TyG index and all-cause mortality in females and CVD mortality in males. It is important to note that these findings may vary due to factors such as insulin resistance, age-related comorbidities, biological aging, and the heterogeneity of the patient population. A recent meta-analysis found no significant connection between the general population's TyG index and all-cause/CVD mortality. However, these results were deemed to be of low reliability^44^.

Subgroup analysis showed that HbA1c and smoking also affected the TyG index and all-cause and CVD deaths. Brown et al.^45^ found that T2D patients with HbA1c > 8.0% had higher all-cause and CVD mortality. The same significant association was observed in the present study at HbA1c ≥ 6.5%. A recent study^46^ showed that smoking significantly increases the risk of IR among adults. Our subgroup study yielded similar findings. An elevated TyG index was linked to a higher risk of death from all causes and from CVD in people with smokers.

IR is present at all stages of T2D development, making it a crucial pathogenic mechanism in the disease^47^. Calcaterr et al.^48^ discovered a strong correlation between the TyG index and HOMA-IR when evaluating IR. It has been reported in recent research^49,50^ that TyG, a novel index for measuring IR, may be more accurate than HOMA-IR in predicting the risk of T2D and related metabolic illnesses. Therefore, early monitoring of TyG may positively impact the prevention of related metabolic diseases. In a recent study, Tian et al.^51^ found that early TyG index buildup was related to a higher risk of CVD and all-cause death than late TyG accumulation, possibly due to continued high TyG exposure. Furthermore, the article states that even lowering TyG levels later in life does not reverse the risk associated with early TyG index accumulation. Therefore, monitoring the TyG index early in life and timely modulation may allow the population to gain long-term survival value.

The study has some strong points. First, the study population was from a nationally representative NHANES population, and 2998 patients with T2D were included, which is a relatively large sample size, and this study enriches the field. Second, this is the first study to examine whether the TyG index is associated with an increased mortality risk in T2D. Third, this research built a sensitivity analysis model and performed a relatively in-depth stratified analysis to guarantee the dependability of the results. Nonetheless, there remain certain limitations in the research. First, there was only one baseline reading for the TyG index. Since it did not reflect participants' long-term TyG index status during follow-up, it may have underestimated the link between the TyG index and mortality risk. Second, the research data come from the United States, and the extrapolation of the study conclusions may be poor due to dietary patterns, living environment, race, etc. Third, although this study corrected for as many relevant confounders as possible, it still failed to exclude potential confounders completely.

## Conclusion

This is the first study to explore the U-shaped connection between the TyG index and death in US middle-aged and senior T2D patients. Over the threshold value, the TyG index was strongly associated with mortality. Therefore, we recommend adding the TyG index to the daily monitoring of diabetic patients and actively managing it to improve the clinical prognosis of middle-aged and elderly diabetic patients.

### Supplementary Information

Supplementary Tables.

## Data Availability

A complete list of the datasets used in this study is available on the NHANES 1999–2018 website (https://wwwn.cdc.gov/nchs/nhanes).
