# Supplementary material for: Triglyceride glucose index as a predictor of mortality in middle-aged and elderly patients with type 2 diabetes in the US
Source: Sci Rep. 2023 Sep 30;13:16478. doi: 10.1038/s41598-023-43512-0 (PMC10542790; doi:10.1038/s41598-023-43512-0)
Supplement: Supplementary file 1 — Supplementary Tables. [file 41598_2023_43512_MOESM1_ESM.docx]

**Supplementary Table 1.** Comparison of Baseline Characteristics of Still-Living Participants and Deceased Participants

|  | **Total** | **Still alive** | **Deceased** | **P-value** |
| --- | --- | --- | --- | --- |
|  |  |  |  |  |
| **N** | 2998 | 2115 | 883 |  |
| **Age, years, Median (IQR)** | 64.00 (45.00-85.00) | 62.00 (54.00, 69.00) | 71.00 (63.00, 79.00) | < 0.001 |
| **Gender, n (%)** |  |  |  | <0.001 |
| Male | 1594 (53.17%) | 1081 (51.11%) | 513 (58.10%) |  |
| **Education status, n (%)** | |  |  | <0.001 |
| Less than high school | 1190 (39.69%) | 769 (36.36%) | 421 (47.68%) |  |
| High school or equivalent | 680 (22.68%) | 466 (22.03%) | 214 (24.24%) |  |
| College or above | 1128 (37.63%) | 880 (41.61%) | 248 (28.09%) |  |
| **Race, n (%)** | |  |  | <0.001 |
| Non-Hispanic White | 635 (21.18%) | 471 (22.27%) | 164 (18.57%) |  |
| Non-Hispanic Black | 311 (10.37%) | 265 (12.53%) | 46 (5.21%) |  |
| Mexican American | 1005 (33.52%) | 587 (27.75%) | 418 (47.34%) |  |
| Other | 1047 (34.92%) | 792 (37.45%) | 255 (28.88%) |  |
| **PIR level, n (%)** | |  |  | <0.001 |
| ≤1.0 | 683 (22.78%) | 467 (22.08%) | 216 (24.46%) |  |
| 1.1–3.0 | 1412 (47.10%) | 947 (44.78%) | 465 (52.66%) |  |
| >3.0 | 903 (30.12%) | 701 (33.14%) | 202 (22.88%) |  |
| **BMI, kg/m2, n (%)** |  |  |  | <0.001 |
| Normal weight | 514 (17.14%) | 320 (15.13%) | 194 (21.97%) |  |
| Low weight | 16 (0.53%) | 8 (0.38%) | 8 (0.91%) |  |
| Overweight | 1026 (34.22%) | 710 (33.57%) | 316 (35.79%) |  |
| Obesity | 1442 (48.10%) | 1077 (50.92%) | 365 (41.34%) |  |
| **Smoking, n (%)** | |  |  | <0.001 |
| Yes | 1549 (51.67%) | 1032 (48.79%) | 517 (58.55%) |  |
| **Alcohol intake, n (%)** |  |  |  | 0.002 |
| Yes | 1967 (65.61%) | 1424 (67.33%) | 543 (61.49%) |  |
| **HF, n (%)** |  |  |  | <0.001 |
| Yes | 302 (10.07%) | 141 (6.67%) | 161 (18.23%) |  |
| **CHD, n (%)** | |  |  | <0.001 |
| Yes | 319 (10.64%) | 169 (7.99%) | 150 (16.99%) |  |
| **Angina, n (%)** | |  |  | <0.001 |
| Yes | 220 (7.34%) | 120 (5.67%) | 100 (11.33%) |  |
| **Heart attack, n (%)** | |  |  | <0.001 |
| Yes | 349 (11.64%) | 181 (8.56%) | 168 (19.03%) |  |
| **Stroke, n (%)** |  |  |  | <0.001 |
| Yes | 257 (8.57%) | 136 (6.43%) | 121 (13.7%) |  |
| **Hypertension, n (%)** | | |  | 0.043 |
| Yes | 1997 (66.61%) | 1385 (65.48%) | 612 (69.31%) |  |
| **Hyperlipidemia, n (%)** | | |  | 0.167 |
| Yes | 1789 (59.67%) | 1279 (60.47%) | 510 (57.76%) |  |
| **Hypoglycemic drugs use, n (%)** | | | | 0.452 |
| Yes | 1702 (56.77%) | 1210 (57.21%) | 492 (55.72%) |  |
| **Total cholesterol, mg/dl, Median (IQR)** | 183.00 (75.00-460.00) | 182.00 (156.00, 213.00) | 183.00 (155.00, 217.00) | 0.576 |
| **LDL cholesterol, mg/dl, Median (IQR)** | 104.00 (15.00-370.00) | 104.00 (80.00, 131.00) | 104.00 (78.00, 132.00) | 0.692 |
| **HbA1c, %, Median (IQR)** | 6.80 (3.50-18.00) | 6.80 (6.20, 7.80) | 6.80 (6.10, 7.90) | 0.841 |

T2D, Type 2 diabetes; TyG, Triglyceride-glucose; IQR, Interquartile range; PIR, Poverty income ratio; BMI, Body mass index; HF, Heart failure; CHD, Coronary heart disease; LDL, Low density lipoprotein; HbA1c, Glycated hemoglobin A1c

**Supplementary Table 2.** Multiple Cox regression analysis of TyG index with all-cause and cardiovascular mortality after exclusion of missing values at baseline

|  | **No. deaths (%)** | | **Crude** | | **Model 1** | | **Model 2** | | **Model 3** |
| --- | --- | --- | --- | --- | --- | --- | --- | --- | --- |
|  |  | | **HR (95%CI), *p* value** | | **HR (95%CI), *p* value** | | **HR (95%CI), *p* value** | | **HR (95%CI), *p* value** |
| All-cause mortality | | | | | | | | | |
| TyG index |  |  | |  | |  | |  | |
| ≤8.72 | 118 (22.26) | 1(Ref) | | 1(Ref) | | 1(Ref) | | 1(Ref) | |
| 8.72-9.15 | 87 (18.32) | 0.71 (0.53~0.93),0.014 | | 0.64 (0.48~0.85),0.002 | | 0.70 (0.53~0.93),0.014 | | 0.70 (0.53~0.93),0.014 | |
| 9.15-9.65 | 119 (24.09) | 0.85 (0.66~1.10),0.210 | | 0.86 (0.66~1.11),0.247 | | 0.88 (0.68~1.14),0.328 | | 0.82 (0.63~1.08),0.159 | |
| >9.65 | 112 (28.64) | 1.07 (0.83~1.39),0.590 | | 1.29 (0.99~1.68),0.063 | | 1.30 (0.99~1.70),0.056 | | 1.07 (0.78~1.48),0.682 | |
| P for trend |  | 0.425 | | 0.034 | | 0.043 | | 0.849 | |
| **Cardiovascular mortality** | | | | | | | | | |
| **TyG index** |  |  | |  | |  | |  | |
| ≤8.72 | 40 (7.55) | 1(Ref) | | 1(Ref) | | 1(Ref) | | 1(Ref) | |
| 8.72-9.15 | 27 (5.68) | 0.64 (0.39~1.04),0.074 | | 0.58 (0.35~0.95),0.029 | | 0.64 (0.39~1.07),0.087 | | 0.61 (0.36~1.01),0.054 | |
| 9.15-9.65 | 35 (7.09) | 0.73 (0.46~1.15),0.171 | | 0.70 (0.44~1.11),0.130 | | 0.76 (0.47~1.21),0.242 | | 0.63 (0.38~1.02),0.059 | |
| >9.65 | 34 (8.70) | 0.95 (0.60~1.50),0.821 | | 1.13 (0.71~1.80),0.611 | | 1.10 (0.68~1.76),0.706 | | 0.66 (0.37~1.19),0.171 | |
| P for trend |  | 0.855 | | 0.635 | | 0.708 | | 0.125 | |

TyG, Triglyceride-glucose index; HR, Hazard ratio; CI, Confidence interval; Ref, Reference

Crude: unadjusted;

Model 1: corrected for age, sex, education level, PIR and BMI;

Model 2: Model 1 + race/ethnicity, smoking status, alcohol consumption status and CVD (including coronary heart disease, heart failure, heart attack, stroke and angina);

Model 3: Model 2 + hypertension, hyperlipidemia, glucose-lowering medication use, HbA1c, LDL-C and TC.

**Supplementary Table 3.** Multiple Cox regression analysis of all-cause and cardiovascular mortality after excluding those taking omega-3 supplements and glucocorticoid drugs

|  | **No. deaths (%)** | **Crude** | **Model 1** | **Model 2** | **Model 3** |
| --- | --- | --- | --- | --- | --- |
|  |  | **HR (95%CI), *p* value** | **HR (95%CI), *p* value** | **HR (95%CI), *p* value** | **HR (95%CI), *p* value** |
| **All-cause mortality** | | | | | |
| **TyG index** |  |  |  |  |  |
| ≤8.72 | 202(27.04) | 1(Ref) | 1(Ref) | 1(Ref) | 1(Ref) |
| 8.72-9.15 | 186(25.27) | 0.77(0.63,0.94),0.011 | 0.76(0.62,0.93),0.007 | 0.79(0.64,0.96),0.021 | 0.80(0.65,0.98),0.028 |
| 9.15-9.65 | 226(30.09) | 0.87(0.72,1.05),0.144 | 0.87(0.72,1.06),0.172 | 0.86(0.71,1.04),0.124 | 0.87(0.71,1.06),0.168 |
| >9.65 | 263(35.64) | 0.97(0.81,1.17),0.777 | 1.11(0.92,1.34),0.286 | 1.15(0.95,1.39),0.166 | 1.13(0.91,1.41),0.252 |
| P for trend |  | 0.740 | 0.089 | 0.072 | 0.186 |
| Cardiovascular mortality | | | | | |
| TyG index |  |  |  |  |  |
| ≤8.72 | 67 (8.97) | 1(Ref) | 1(Ref) | 1(Ref) | 1(Ref) |
| 8.72-9.15 | 52 (7.07) | 0.65(0.45,0.94),0.020 | 0.63(0.44,0.91),0.014 | 0.69(0.48,1.00),0.050 | 0.69(0.47,1.00),0.048 |
| 9.15-9.65 | 62 (8.26) | 0.72(0.51,1.01),0.058 | 0.71(0.50,1.00),0.051 | 0.73(0.51,1.04),0.083 | 0.72(0.50,1.03),0.075 |
| >9.65 | 84 (11.38) | 0.94(0.68,1.29),0.688 | 1.05(0.75,1.45),0.786 | 1.12(0.80,1.58),0.497 | 1.04(0.71,1.53),0.837 |
| P for trend |  | 0.995 | 0.521 | 0.373 | 0.816 |

TyG, Triglyceride-glucose index; HR, Hazard ratio; CI, Confidence interval; Ref, Reference

Crude: unadjusted;

Model 1: corrected for age, sex, education level, PIR and BMI;

Model 2: Model 1 + race/ethnicity, smoking status, alcohol consumption status and CVD (including coronary heart disease, heart failure, heart attack, stroke and angina);

Model 3: Model 2 + hypertension, hyperlipidemia, glucose-lowering medication use, HbA1c, LDL-C and TC.

**Supplementary Table 4.** Multiple Cox regression analysis of all-cause and cardiovascular mortality after adjustment for the GNRI

|  | **No. deaths (%)** | **Crude** | **Model 1** | **Model 2** | **Model 3*** |
| --- | --- | --- | --- | --- | --- |
|  |  | **HR (95%CI), *p* value** | **HR (95%CI), *p* value** | **HR (95%CI), *p* value** | **HR (95%CI), *p* value** |
| **All-cause mortality** | | | | | |
| **TyG index** |  |  |  |  |  |
| ≤8.72 | 203(26.92) | 1(Ref) | 1(Ref) | 1(Ref) | 1(Ref) |
| 8.72-9.15 | 186(25.14) | 0.77(0.63~0.94),0.011 | 0.76(0.62~0.93),0.007 | 0.79(0.65~0.97),0.022 | 0.81(0.66~0.99),0.038 |
| 9.15-9.65 | 229(30.25) | 0.88(0.73~1.06),0.175 | 0.88(0.73~1.07),0.200 | 0.87(0.72~1.06),0.161 | 0.90(0.74~1.10),0.295 |
| >9.65 | 265(35.48) | 0.97(0.80~1.16),0.718 | 1.09(0.91~1.32),0.349 | 1.14(0.94~1.38),0.172 | 1.17(0.94~1.45),0.158 |
| P for trend |  | 0.776 | 0.108 | 0.070 | 0.102 |
| **Cardiovascular mortality** | | | | | |
| **TyG index** |  |  |  |  |  |
| ≤8.72 | 67(8.89) | 1(Ref) | 1(Ref) | 1(Ref) | 1(Ref) |
| 8.72-9.15 | 52(7.03) | 0.65(0.46~0.94),0.022 | 0.64(0.44~0.92),0.015 | 0.70(0.48~1.01),0.055 | 0.70(0.48~1.01),0.056 |
| 9.15-9.65 | 62(8.19) | 0.72(0.51~1.01),0.060 | 0.71(0.50~1.00),0.051 | 0.74(0.52~1.05),0.091 | 0.73(0.51~1.05),0.093 |
| >9.65 | 84(11.24) | 0.93(0.67~1.28),0.644 | 1.03(0.74~1.43),0.851 | 1.12(0.80~1.57),0.506 | 1.05(0.72~1.56),0.788 |
| P for trend |  | 0.943 | 0.575 | 0.380 | 0.762 |

TyG, Triglyceride-glucose index; HR, Hazard ratio; CI, Confidence interval; Ref, Reference; GNRI, Geriatric Nutrition Index

Crude: unadjusted;

Model 1: corrected for age, sex, education level, PIR and BMI;

Model 2: Model 1 + race/ethnicity, smoking status, alcohol consumption status and CVD (including coronary heart disease, heart failure, heart attack, stroke and angina);

Model 3*: Model 2 + hypertension, hyperlipidemia, glucose-lowering medication use, HbA1c, LDL-C, TC and GNRI.
